# Examining Health Outcomes in Juvenile Idiopathic Arthritis: A Genetic Epidemiology Study

**DOI:** 10.1002/acr2.11404

**Published:** 2022-01-25

**Authors:** Sarah L. N. Clarke, Rebecca C. Richmond, Jie Zheng, Wes Spiller, Athimalaipet V. Ramanan, Gemma C. Sharp, Caroline L. Relton

**Affiliations:** ^1^ Medical Research Council Integrative Epidemiology Unit, University of Bristol Bristol UK; ^2^ School of Population Health Sciences, University of Bristol Bristol UK; ^3^ Department of Paediatric Rheumatology, Bristol Royal Hospital for Children Bristol UK; ^4^ School of Translation Health Sciences, University of Bristol Bristol UK

## Abstract

**Objective:**

Juvenile idiopathic arthritis (JIA) is the most common pediatric rheumatic disease; however, little is known about its wider health impacts. This study explores health outcomes associated with JIA genetic liability.

**Methods:**

We used publicly available genetic data sets to interrogate the genetic correlation between JIA and 832 other health‐related traits using linkage disequilibrium score regression. Two‐sample Mendelian randomization (2SMR) was used to examine four genetic correlates for evidence of causality.

**Results:**

We found robust evidence (adjusted *P* [*P*_adj_] < 0.05) of genetic correlation between JIA and rheumatoid arthritis (genetic correlation [*r*_g_] = 0.63, *P*_adj_ = 0.029), hypothyroidism/myxedema (*r*_g_ = 0.61, *P*_adj_ = 0.041), celiac disease (CD) (*r*_g_ = 0.58, *P*_adj_ = 0.032), systemic lupus erythematosus (*r*_g_ = 0.40, *P*_adj_ = 0.032), coronary artery disease (CAD) (*r*_g_ = 0.42, *P*_adj_ = 0.006), number of noncancer illnesses (*r*_g_ = 0.42, *P*_adj_ = 0.016), paternal health (*r*_g_ = 0.57, *P*_adj_ = 0.032), and strenuous sports (*r*_g_ = −0.52, *P*_adj_ = 0.032). 2SMR analyses found robust evidence that genetic liability to JIA was causally associated with the number of noncancer illnesses reported by UK Biobank (UKBB) participants (increase of 0.03 noncancer illnesses per doubling odds of JIA, 95% confidence interval 0.01‐0.05).

**Conclusion:**

This study illustrates genetic sharing between JIA and a diversity of health outcomes. The causal association between genetic liability to JIA and noncancer illnesses suggests a need for broader health assessments of patients with JIA to reduce their potential comorbid burden. The strength of genetic correlation with hypothyroidism and CD implies that patients with JIA may benefit from CD and thyroid function screening. Strong positive genetic correlation between JIA and CAD supports the need for cardiovascular risk assessment and risk factor modification.

**Significance & Innovations**

- This study demonstrates the causal role of genetic liability to juvenile idiopathic arthritis (JIA) on comorbidity and the potential breadth and burden of JIA‐associated comorbidity later in life.
- JIA was genetically correlated with multiple other autoimmune traits, including hypothyroidism and celiac disease; thus, patients may benefit from routine screening of these conditions.
- JIA is genetically correlated with both coronary artery disease and a number of adverse cardiometabolic traits, highlighting the need to consider cardiovascular risk factor assessment and modification in JIA.

## INTRODUCTION

Juvenile idiopathic arthritis (JIA) is the most common rheumatic disease of childhood, with an estimated prevalence of 32.6 per 100,000 in Europeans (1). Approximately 40% of patients with JIA continue to have active disease and/or require medication into adulthood (2). As increasing numbers of pediatric rheumatology patients transition into adult services, there is a need to understand adult outcomes of pediatric rheumatic diseases that may be related to treatments, active disease, or other comorbidity (3). Improving our understanding of JIA‐associated comorbidity is important for patient counseling and treatment strategy, particularly as patients with JIA become more autonomous in their behaviors. Observational studies of adult outcomes of rare pediatric‐onset conditions, although important, are challenging.

The increasing availability of genetic data in pediatric rheumatology plus novel methods to probe association and causality complement traditional observational epidemiology and provide additional scope to investigate adult health outcomes in JIA.

**FIGURE 1:**
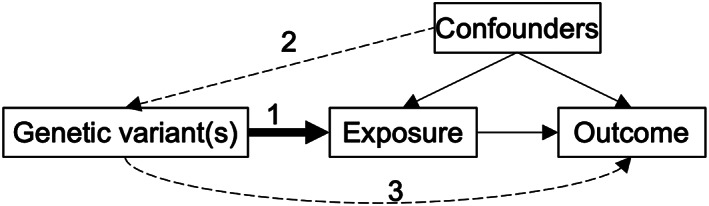
Direction acyclic graph representing Mendelian randomization (MR). To be valid MR instruments, genetic variants must be strongly associated with the exposure (1), cannot be associated with confounders of the exposure or the outcome (2), and cannot influence the outcome except via the exposure (3).

Genetic epidemiology methods, such as linkage disequilibrium score regression (LDSC) and Mendelian randomization (MR), enable estimation of the genetic and causal relationships between JIA and other health outcomes. LDSC uses genome‐wide association study (GWAS) summary statistics to estimate the single‐nucleotide polymorphism (SNP) heritability of a trait (4) and genetic correlation (ie, genetic sharing) between two traits (5). Cross‐trait LDSC provides an efficient hypothesis‐free screen of correlated traits, whereas MR is a more robust approach to estimate causal relationships between correlated traits (6). Two‐sample MR (2SMR) uses genetic variants (SNPs) from summary statistics of separate GWAS samples (one for the exposure and one for the outcome) within a single analysis (7). This increases the number of trait combinations that can be examined and substantially boosts the sample size and, therefore, power of these analyses. SNPs identified as genetic instruments for the exposure are extracted from the GWAS summary statistics of the selected outcomes. To produce unbiased results, SNPs used in MR must be robustly associated with the exposure (Figure 1), not be associated with confounders of the exposure and outcome, and must only be associated with the outcome via the exposure (ie, no horizontal pleiotropy). A range of methods and sensitivity analyses can be applied to evaluate these MR assumptions.

The aims of this study were, firstly, to use a hypothesis‐free approach to examine the shared genetic correlations of JIA with other health‐related traits using cross‐trait LDSC and, secondly, to use 2SMR to interrogate whether JIA is causally associated with genetically correlated health‐related traits.

## Materials and methods

### Genetic correlation

**FIGURE 2:**
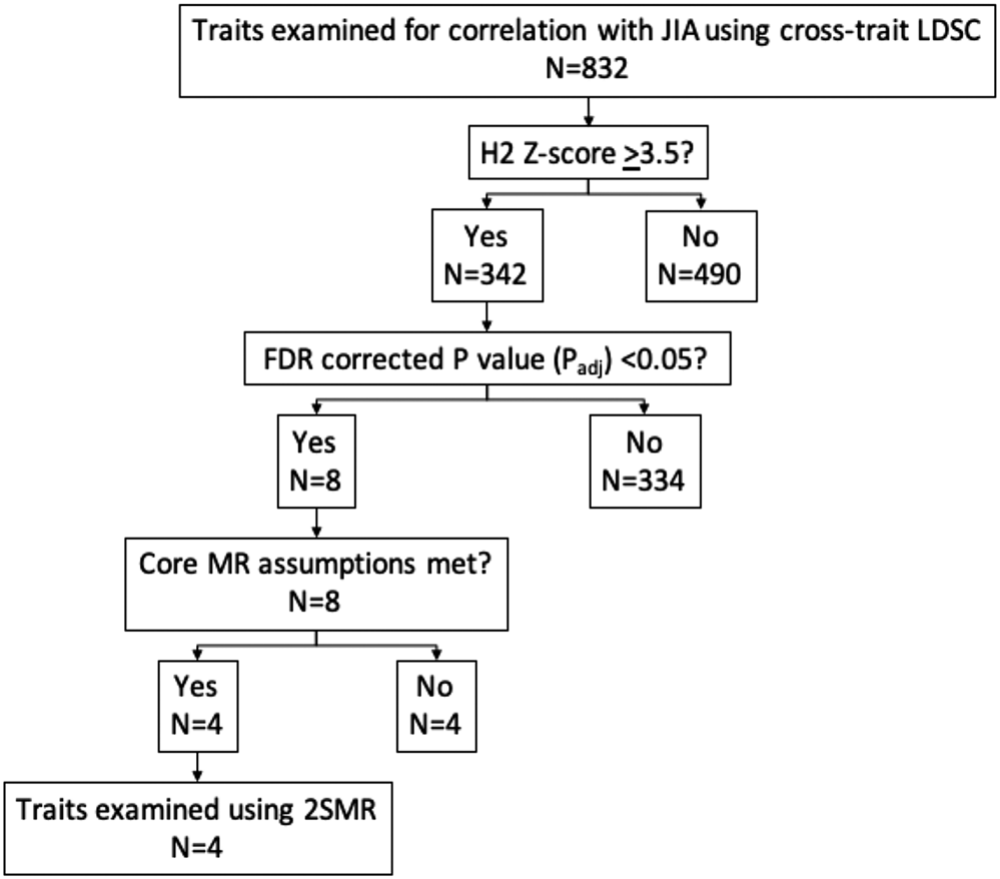
Study design of this genetic epidemiology study. adj, adjusted; FDR, false discovery rate; JIA, juvenile idiopathic arthritis; LDSC, linkage disequilibrium score regression; MR, Mendelian randomization; 2SMR, two‐sample Mendelian randomization.

To estimate genetic correlation between JIA and 832 other health‐related traits, we used cross‐trait LDSC implemented in the LD Hub (version 1.9.0) online platform (see Figure 2 for analysis pipeline) (5, 8). Single‐trait LDSC exploits the expected relationship between the SNP association with the trait of interest and the degree of linkage disequilibrium (LD) between SNPs, whereby association test statistics of SNPs are regressed against their LD scores. SNPs that tag more of the genome (ie have high LD scores) have a higher likelihood of tagging a causal variant and thus have larger effect sizes (4). By extension, cross‐trait LDSC estimates genetic correlation (*r*_g_) between traits by regressing the product of the *z* scores from two studies of traits against the LD score for each SNP, with possible *r*_g_ estimates ranging from −1 to 1 (5).

GWAS summary data held within LD Hub are not sex stratified and are predominantly from populations of European ancestry. All studies in LD Hub have undergone rigorous quality control (8). The JIA data set comprised a fine‐mapping study of 2,816 oligoarticular and rheumatoid‐factor‐negative (RF^−^) patients with polyarticular JIA (76.6% female) and 13,056 healthy controls (55.1% female) of European ancestry (9). Data sets for other traits within LD Hub ranged in sample sizes from 5,422 to 337,199 (Supplementary Table 1). As per LD Hub defaults, SNPs from the major histocompatibility complex (MHC) region were removed because of complex LD and potentially extreme effect sizes (8). Traits with evidence of low heritability (*H* ^2^ *z* score <3.5) were excluded. Benjamini–Hochberg (10) adjustment was applied to account for 832 individual tests; an adjusted *P* value (*P*_adj_) <0.05 was considered statistically significant. For clarity, some traits have been renamed; Supplementary Table 2 provides a full index of traits, definitions, and references.

### 2SMR

#### Selection of genetic instruments of JIA

The JIA instruments were selected from the same JIA data set used previously (9). We selected conditionally independent (LD *r* ^2^ < 0.001) variants that were associated with JIA (*P* < 5 × 10^−8^) in an additive model as instruments for MR. The variance explained (*R* ^2^) and *F* statistic were calculated for the JIA instrument (see Supplementary Methods).

#### Outcome selection for MR analysis

Of the 832 traits examined, eight passed the Benjamini–Hochberg adjusted threshold (*P*_adj_ < 0.05) in relation to their genetic correlation with JIA (see Results). We further removed four autoimmune traits because the shared immunogenetics of many autoimmune diseases was likely to violate the third assumption of MR, ie, that of no genetic pleiotropy (Figure 1). The remaining four traits were used as outcomes for 2SMR. Supplementary Table 3 details the GWAS data sets used in this analysis.

#### Discovery MR analysis

Evidence for a causal relationship between JIA and selected outcomes was explored using 2SMR implemented using the TwoSampleMR package (11) in the R software platform (version 1.2.5019; R Foundation for Statistical Computing) (12). The selected JIA instruments were looked up in the outcome GWAS data sets from the MR Base database (11). LD proxies (*r* ^2^ > 0.8) were used where the instrument was not available in the outcome GWAS. The alleles were harmonized to ensure that the SNP exposure effect and the SNP outcome effect corresponded to the same allele. Palindromic SNPs were aligned using default settings, and noninferable palindromic SNPs were excluded to limit effect allele ambiguity between JIA and outcome data sets. We applied the inverse variance weighted (IVW) method to estimate the effect of JIA on selected outcomes. Briefly, the IVW method calculates a weighted average of effect estimates obtained using each individual SNP (via a Wald ratio) through a fixed‐effect meta‐analysis. SNPs are typically weighted by their corresponding inverse variance (13).

#### MR sensitivity analyses

Although the inclusion of additional genetic variants robustly associated with the exposure increases the statistical power of MR analyses, it also increases the likelihood of including pleiotropic SNPs, which violate core assumptions of MR and can lead to imprecise estimates. Observed heterogeneity in effect estimates can be used as an indicator of pleiotropic bias. Heterogeneity was assessed using Cochran's *Q* statistic (14). To further test and account for horizontal pleiotropy, we employed multiple sensitivity analyses using MR‐Egger regression (13), the weighted median estimator (15), Mendelian randomisation pleiotropy residual sum and outlier (MR‐PRESSO) (16), and RadialMR (17). Because these approaches rely on one or more nonoverlapping assumptions, consistent estimates across all methods makes bias due to pleiotropy less likely. Data are presented as *β* coefficients for continuous traits and odds ratios (ORs) for binary traits. To aid interpretation, MR estimates were transformed to represent the change per doubling in odds of JIA. However, given that JIA represents a binary exposure, it may be more straightforward to consider the MR estimates as evidence for the existence versus absence of a causal effect of exposure (JIA) on the selected outcomes (18). For further details of MR methods and data transformations, see Supplementary Methods.

## Results

### Quality control and SNP heritability analysis for JIA

Single‐trait LDSC estimated the JIA SNP *H* ^2^ as 0.58 (SE 0.20). The JIA data set met the recommended thresholds (8) for genetic correlation analysis (Supplementary Table 4).

### Genetic correlations between JIA and health outcomes

**FIGURE 3:**
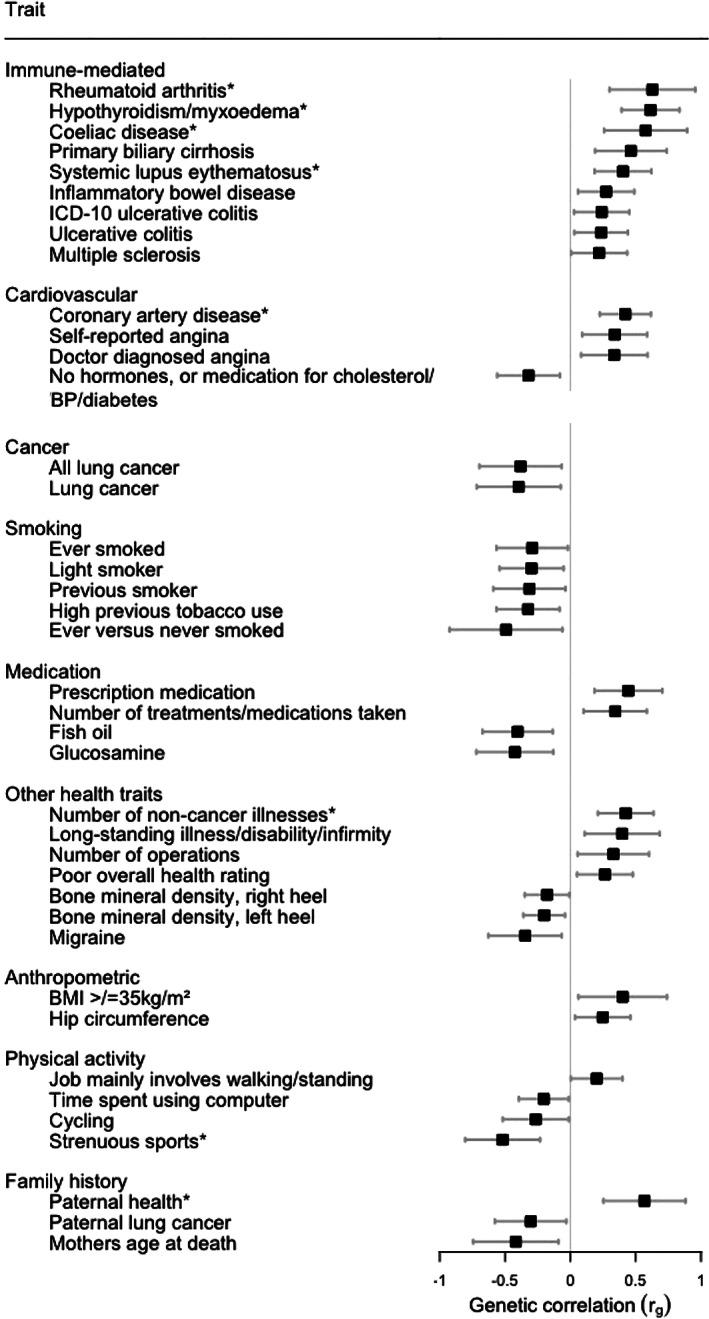
Genetic correlation between juvenile idiopathic arthritis and 40 health‐related traits with *P* < 0.05. *Those traits with a *P* value <0.05 after adjustment for multiple testing (*P*_adj_ < 0.05). BMI, body mass index; BP, blood pressure; ICD‐10, *International Classification of Diseases, 10th Revision*.

We examined the genetic correlation between JIA and 832 traits in LD Hub (Supplementary Table 1). After exclusion of traits for which no correlation estimate was available or heritability *z* scores were low (<3.5), we identified eight health‐related traits that were genetically correlated with JIA after correction for multiple testing (*P*_adj_ < 0.05; n = 8), plus 32 suggestively correlated traits (*P* < 0.05; Figure 3).

Four autoimmune conditions were genetically correlated with JIA after correction for multiple testing (Figure 3): rheumatoid arthritis (RA) (*r*_g_ = 0.63, 95% confidence interval [CI] 0.30‐0.96, *P*_adj_ = 0.029), hypothyroidism/myxedema (*r*_g_ = 0.61, 95% CI 0.39‐0.84, *P*_adj_ = 4.9 × 10^−5^), celiac disease (CD) (*r*_g_ = 0.58, 95% CI 0.26‐0.89, *P*_adj_ = 0.032), and systemic lupus erythematosus (SLE) (*r*_g_ = 0.40, 95% CI 0.19‐0.62, *P*_adj_ = 0.032). A further five autoimmune conditions showed positive correlation with JIA: primary biliary cirrhosis (PBC) (*r*_g_ = 0.46, 95% CI 0.19‐0.74, *P* = 9.0 × 10^−4^), inflammatory bowel disease (*r*_g_ = 0.27, 95% CI 0.06‐0.49, *P* = 0.012), ulcerative colitis (UC) (*r*_g_ = 0.24, 95% CI 0.03‐0.44, *P* = 0.024; *r*_g_ = 0.24, 95% CI 0.03‐0.45, *P* = 0.026) and multiple sclerosis (*r*_g_ = 0.22, 95% CI 0.01‐0.43, *P* = 0.044), although these did not survive adjustment for multiple testing (Figure 3). Atopic disorders (asthma, eczema/dermatitis, hay fever/rhinitis) consistently showed no correlation with JIA (Supplementary Figure 1).

Genetic correlation was also seen between JIA and multiple cardiometabolic traits. A positive correlation was observed between JIA and coronary artery disease (CAD) (*r*_g_ = 0.42, 95% CI 0.22‐0.62, *P*_adj_ = 6.0 × 10^−3^; Figure 3). There was also suggestive positive genetic correlation between JIA and two angina traits (*r*_g_ = 0.34, 95% CI 0.09‐0.59, *P* = 7.40 × 10^−3^ and *r*_g_ = 0.34, 95% CI 0.08‐0.59, *P* = 9.5 × 10^−3^) and negative correlation between JIA and reporting to not take medication for cholesterol, blood pressure, diabetes, or exogenous hormones (*r*_g_ = −0.32, 95% CI −0.56 to −0.08, *P* = 9.0 × 10^−3^). JIA also showed genetic correlation with cardiometabolic risk factors traits, including physical activity, body habitus, and smoking. The only negative correlation with JIA after adjustment for multiple testing was for strenuous sports (*r*_g_ = −0·52, 95% CI −0.80 to −0.23, *P*_adj_ = 0.032). However, suggestive correlation was also seen between JIA and other markers of more sedentary behavior, eg, reporting to have a job involving mainly walking or standing (*r*_g_ = 0.20, 95% CI 0.00‐0.40, *P* = 0.047), bone mineral density (left heel: *r*_g_ = −0.18, 95% CI −0.36 to −0.04, *P* = 0.040; right heel: *r*_g_ = −0.20, 95% CI −0.35 to −0.01, *P* = 0.014), and reporting to use cycling as a mode of transport (*r*_g_ = −0.26, 95% CI −0.52 to −0.01, *P* = 0.040). Suggestive positive genetic correlations were also seen between JIA and anthropometric traits, including obesity (body mass index ≥35: *r*_g_ = 0.40, 95% CI 0.06‐0.74, *P* = 0.02) and hip circumference (*r*_g_ = 0.25, 95% CI 0.04‐0.46, *P* = 0.02).

Furthermore, JIA was genetically correlated with a number of other health traits (Figure 3), including the number of noncancer illnesses (*r*_g_ = 0.42, 95% CI 0.21‐0.64, *P*_adj_ = 0.016) and paternal health (*r*_g_ = 0.57, 95% CI 0.25‐0.88, *P*_adj_ = 0.032), following adjustment for multiple testing. A number of correlations were also seen with other adverse individual and familial health‐related outcomes, including overall assessments of health, although these did not survive correction for multiple testing.

### 2SMR study

#### Associations between JIA and non‐immune‐mediated traits

**FIGURE 4:**
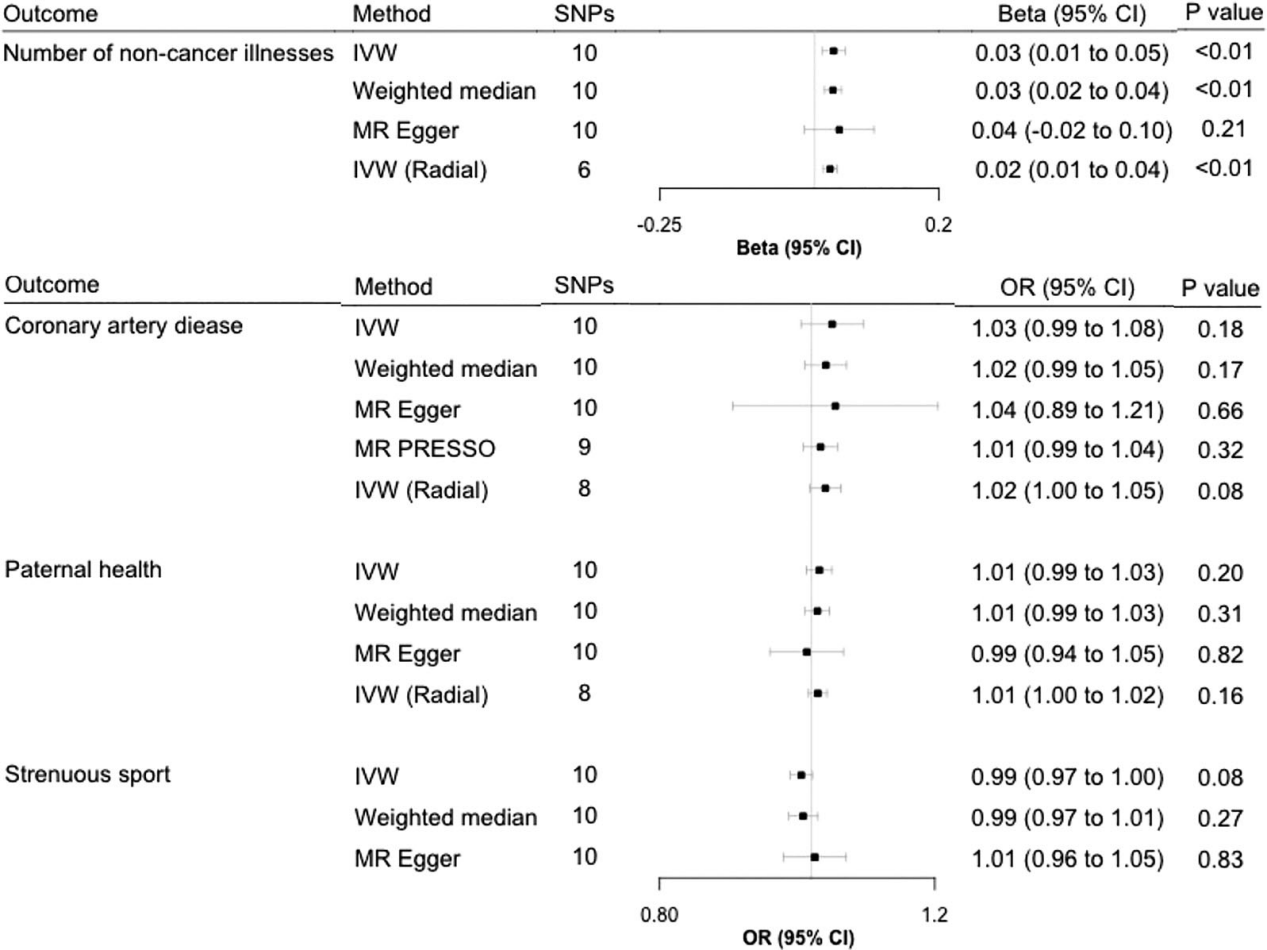
Two‐sample Mendelian randomization (MR) analysis showing the effects of genetically predicted juvenile idiopathic arthritis (JIA) on selected health outcomes. Unadjusted MR‐Egger estimates are reported as $I_{\mathrm{GX}}^{2}$>90% in all analyses. MR‐PRESSO estimates are reported where the distortion test *P* value <0.05. RadialMR estimates are reported where outliers were detected and corrected for. *β* Values are reported for continuous outcomes and interpreted as increases per doubling odds of JIA. Odds ratios (ORs) are reported for binary outcomes and interpreted as ORs for the outcome per doubling in odds of JIA. Full details of all sensitivity analyses can be found in Supplementary Tables 6–10. CI, confidence interval; IVW, inverse variance weighted; MR‐PRESSO, Mendelian randomisation pleiotropy residual sum and outlier; SNP, single‐nucleotide polymorphism.

LDSC identified four nonautoimmune traits strongly genetically correlated with JIA. To explore whether JIA is causally related to these traits, we undertook a 2SMR study. Eleven independent JIA‐associated SNPs were used as instruments (Supplementary Table 5), with appropriate strength for MR analysis (*F* statistic = 52.64, *R* ^2^ = 0.0352). Genetically predicted JIA was causally implicated in the number of reported noncancer illnesses (increase of 0.03 noncancer illnesses per doubling odds of JIA, 95% CI 0.01‐0.05). There was limited evidence of a causal relationship between genetically predicted JIA and CAD (OR 1.03 per doubling odds of JIA, 95% CI 0.99‐1.08), with the estimates from all MR methods directionally consistent (Figure 4). We also found little evidence that genetically predicted JIA is causally associated with strenuous sports (OR 0.98 per doubling odds of JIA, 95% CI 0.97‐1.00) or paternal health (OR 1.03 per doublings odds of JIA, 95% CI 0.98‐1.09) (Figure 4). We also conducted leave‐one‐out analysis, showing that no single SNP was driving these causal estimates (Supplementary Figure 2).

#### Assessment of heterogeneity and control for pleiotropy

Cochran's *Q* showed some evidence of heterogeneity (*P* < 0.05) in all MR analyses, except for the analysis evaluating the effect of JIA on strenuous sports (Supplementary Table 6); thus, pleiotropy was further assessed. There was minimal evidence of pleiotropy (*P* > 0.05) for any of the outcomes based on the MR‐Egger intercept (Supplementary Table 7). The MR‐PRESSO global test was applied to all outcomes, with the distortion test only significant (*P* < 0.05) for CAD (Figure 3, Supplementary Table 8). RadialMR identified outlying SNPs for CAD (number of SNPs [nSNPs] = 2), paternal health (nSNPs = 2), and number of noncancer illnesses (nSNPs = 4); therefore, IVW estimates were recalculated, with outlying SNPs removed from the instrument (Figure 4, Supplementary Table 9). Nevertheless, despite rigorous assessment for heterogeneity and pleiotropy, the causal estimates from these sensitivity analyses were also comparable with the original IVW estimate.

## Discussion

The major aims of this study were to a) identify health‐related traits that are genetically correlated with JIA and b) investigate whether these may indicate a causal effect of JIA to assist in patient counseling, risk stratification, lifestyle modification, and treatment targeting for patients with JIA. We show that genetic liability to JIA is correlated with genetic liability to many systemic and organ‐specific autoimmune and nonautoimmune disorders. We found evidence for a causal effect of JIA on the number of noncancer illnesses reported in adulthood, illustrating the potential comorbidity associated with JIA. There was limited evidence that genetic liability to JIA is causally implicated in CAD, strenuous sports, or paternal health, suggesting that the genetic correlation seen between these traits is more likely due to shared genetic architecture, exposure to environmental risk factors, or other shared factors that influence these traits but via different causal paths. However, the JIA data set has a relatively small sample size and because estimates from multiple MR methods are consistent, we cannot fully exclude the possibility of a small causal effect of liability to JIA on these outcomes.

JIA is reported to be one of the most highly heritable pediatric autoimmune conditions, with heritability estimates ranging from 53% to 73% (19, 20). Similarly, we report JIA SNP *H* ^2^ as 58% using LDSC. Previous studies into the genetic architecture of pediatric autoimmune disorders report genetic sharing between JIA and a number of immune‐mediated disorders using genome‐wide and locus‐specific analyses, including CD, UC, PBC, psoriasis, type 1 diabetes (T1D), and ankylosing spondylitis (19, 21). Correspondingly, we identified strong evidence of genetic correlation between JIA and CD plus suggestive evidence of correlation with UC and PBC. T1D (proxied by “started insulin within one year of diagnosis of diabetes”) and ankylosing spondylitis were excluded from our analyses because of low heritability, although the direction of association agrees with previous reports. Psoriasis was positively correlated with JIA in our study, but this association did not survive multiple testing (Supplementary Table 1). The JIA data set (9) used in this study excluded psoriatic and enthesitis‐related arthritis subtypes of JIA, potentially masking or attenuating any association between psoriasis and spondyloarthropathies. We found novel evidence of significant genetic correlation with hypothyroidism/myxedema and SLE, in keeping with suggestive evidence from smaller cohorts (19, 21).

This study found cumulative evidence that JIA is associated with cardiovascular disease (CVD) risk. CAD (22) was the nonautoimmune trait most strongly genetically correlated with JIA, a finding supported by UKBB data (23) showing suggestive positive genetic correlation between JIA and angina and suggestive negative correlation between JIA and absence of medications used in cardiovascular risk reduction. Furthermore, JIA was suggestively genetically correlated with several cardiometabolically unfavorable risk factors (obesity, hip circumference, and sedentary behavior).

Observationally, JIA is associated with poor health outcomes in adulthood (24, 25, 26). Accordingly, we report positive genetic correlation between JIA and number of noncancer illness, longstanding disability/infirmity, number of operations, and poorer overall health rating. Smoking is generally associated with increased risk of autoimmunity (27); thus, it is interesting to note that in our study, this association is in the opposite direction. Explanations may include the temporal relationship between JIA onset and smoking initiation, selection bias of participants in the outcome cohorts, or modification of social behaviors as a result of underlying ill health; further work to examine these associations is required.

Our findings highlight a number of potential clinical implications; however, we caution that further investigation is warranted. The causal association between liability to JIA and number of noncancer illnesses suggests a need for risk assessment and risk factor modification in patients with JIA to reduce the burden of its comorbidity. In the United Kingdom, children with T1D are recommended to have regular assessment of thyroid function (28). Similarly, serological screening for CD is recommended in autoimmune thyroid disease (29). Given the genetic correlation between JIA and both hypothyroidism/myxedema and CD shown here, our findings support the hypothesis that patients with JIA are also at increased risk of organ‐specific autoimmunity. The shared immunogenetics of autoimmune disease likely violates the third assumption of MR; thus, examining a causal role for JIA on other autoimmune disorders is outside the scope of this study. Nevertheless, further assessment of whether thyroid function testing and CD screening clinically benefits patients with JIA and should be included in standards of care is warranted. Furthermore, extending the knowledge of shared genetic architecture between JIA and other autoimmune traits builds on our understanding of etiopathogenesis of autoimmunity and may represent an opportunity to repurpose drugs or therapeutic strategies for JIA.

There is growing recognition of the interplay between CVD and autoimmune conditions. Patients with RA are at 50% increased risk of CVD versus patients without RA, forming the basis for European guidance on the assessment and management of CVD risk in RA (30). However the association between RA and CAD is apparently not driven by shared genetic influences (LDSC: *r*_g_ = −0.063, *P* = 0.44) (5). Patients with JIA are hypothesized to have increased CVD risk compared with patients with RA due to the childhood onset of disease and, thus, prolonged duration of inflammation (31), and impaired vascular endothelial function during childhood has been demonstrated in JIA (32). Given their phenotypic similarity, JIA–RA was unsurprisingly the strongest observed genetic correlation. Our finding that JIA, unlike RA, shows significant positive correlation with CAD suggests underlying shared genetic factors between JIA and CAD that are not found between RA and CAD, upholding the concern of additional CVD risk in JIA. Furthermore, we present suggestive evidence of genetic correlation between JIA and several cardiometabolically unfavorable traits. Although we did not demonstrate strong evidence that JIA is causal for CAD, the MR estimate was not null, and given the wide CIs, this likely represents limited power to detect small causal effects using the available data sets. Nevertheless, clinically, these findings support robust CVD risk factor assessment and modification in JIA, although the optimal nature and timing of such intervention requires further study.

The main strength of this study is its hypothesis‐free approach to capture a broad range of JIA‐associated health outcomes. The outcome data sets used in this study have considerably larger sample sizes than those used in previous JIA studies (19, 21), often with higher case fractions, substantially increasing the power to detect associations. Reporting of results based on adjusted and unadjusted levels of significance limits the risk of false‐positive results while capturing potentially clinically important findings. Concordant genetic correlation results across presumed nonoverlapping data sets add confidence to our findings. For 2SMR analysis, we undertook rigorous selection of SNPs to instrument JIA. The use of multiple MR methods, each reporting similar effect estimates with overlapping CIs, adds credibility and assurance to our results and limits spurious associations due to horizontal pleiotropy and LD.

The main limitations of this study lie with the data sets. The JIA data set originates from a 2013 Immunochip study, which limits the number of SNPs and genomic coverage available and may affect LDSC estimates. However the exclusion of the MHC region reduces the likelihood of spurious genetic correlations. Replication and validation of our findings using a JIA data set with broader genomic coverage and a larger sample size would be valuable, particularly within the 2SMR context. The JIA data set maximizes study homogeneity by including only oligoarticular and RF^−^ polyarticular subtypes (9). Thus, we cannot delineate whether our findings are subtype specific or pan‐JIA. Several UKBB traits derive from self‐reported data in which reliability is less certain and case fractions are generally lower compared with case‐control studies that are enriched for physician‐diagnosed cases. Nevertheless, genetic correlation estimates are consistent for traits (eg, UC) in which both self‐reported and doctor‐diagnosed/*International Classification of Diseases, 10th Revision* coded variables are available. Some traits derive from mixed ancestry, rather than exclusively European, populations (Supplementary Table 1), which can lead to departure from LD structure and bias results. We did not exclude traits with mixed ancestry, provided the data set was predominantly European. It is unlikely this had a substantial impact on our findings; CAD assessed using the CardiogramC4D data set (22) (77% European) and angina traits from the UKBB (23) (100% European) have the same direction and similar magnitude of effect. We used GWAS summary level data, and therefore we cannot account for other sources of bias (eg, cohort selection). In the context of 2SMR, this also means that we cannot formally test assumption two (that genetic variants are not associated with confounders of the exposure or the outcome; Figure 1) because the lack of access to individual‐level data precludes assessment of the study‐specific (sample‐specific) confounding structures, such as population stratification. However, our study includes only data sets that have been derived exclusively from European populations and that have also been adjusted for genetic principal components, minimizing the risk of violating this assumption. The JIA data set derives from children, whereas the outcome GWAS data sets used derive primarily from adults; therefore, we assume the effect of JIA‐associated genetic variants is constant over the life course. Finally, given the low population prevalence of JIA, there are likely few cases within the outcome GWAS data sets; thus, we can only infer causal effects of genetically predicted JIA on other traits.

This study demonstrates causal evidence of a comorbid burden of JIA. We show JIA is genetically correlated with several novel and important long‐term health outcomes, particularly CAD and other systemic and organ‐specific autoimmune disorders. 2SMR suggests the association between JIA and CAD is more likely one of correlation rather than causation. Nevertheless, our data support the initiation of CVD risk assessment and management guidelines for patients with JIA, alongside consideration of thyroid function monitoring and CD screening. To our knowledge, our study applies these techniques to JIA for the first time and illustrates the power of harnessing such methods to leverage genetic data for patient benefit. Corroboration of these findings using genome‐wide data, including all JIA subtypes, is needed.

## AUTHOR CONTRIBUTIONS

All authors were involved in drafting the article or revising it critically for important intellectual content, and all authors approved the final version to be published. Dr. Clarke and Dr Zheng had full access to all of the data in the study and takes responsibility for the integrity of the data and the accuracy of the data analysis.

### Study conception and design

Clarke.

### Acquisition of data

Clarke, Zheng.

### Analysis and interpretation of data

Clarke, Richmond, Zheng, Spiller, Ramanan, Sharp, Relton.

## Supporting information

Disclosure Form

**Supplementary Figure 1** Genetic correlation between JIA and 22 immune‐mediated traits. Black boxes indicate Padj <0.05 after adjustment for multiple testing, dark grey boxes indicate unadjusted P < 0.05 and light grey boxes indicate unadjusted P > 0.05. Method of ascertainment of UK Biobank variables is indicated by superscript ‐*self‐reported data, ^ICD‐10 coded data, #doctor diagnosed data or ~ diagnosed by doctor. Data source provided in parentheses for each trait (see Supplementary Table 1 for more details). UKBB, UK Biobank.
**Supplementary figure 2**: Leave one out analysis. In order to test whether any individual SNPs were driving the association between JIA and (A) coronary artery disease, (B) strenuous sports, (C) paternal health and (D) number of non‐cancer illnesses, a leave on out analysis was conducted. Each row represents 2SMR analysis of JIA on the selected outcome using all SNPs in the JIA instrument, except the SNP listed on the y‐axis. The point represents the MR effect size (beta) and the bars represent 95% confidence intervals. The row in red represents the estimate when all SNPs are included in the analysis.

**Supplementary S1** Methods

Supplementary Tables S1
