# Supplementary material for: Examining Health Outcomes in Juvenile Idiopathic Arthritis: A Genetic Epidemiology Study
Source: ACR Open Rheumatol. 2022 Jan 25;4(4):363–70. doi: 10.1002/acr2.11404 (PMC8992462; doi:10.1002/acr2.11404)
Supplement: Supplementary file 2 — Supplementary Figure 1 Genetic correlation between JIA and 22 immune‐mediated traits. Black boxes indicate Padj <0.05 after adjustment for multiple testing, dark grey boxes indicate unadjusted P < 0.05 and light grey boxes indicate unadjusted P > 0.05. Method of ascertainment of UK Biobank variables is indicated by superscript ‐*self‐reported data, ^ICD‐10 coded data, #doctor diagnosed data or ~ diagnosed by doctor. Data source provided in parentheses for each trait (see Supplementary Table 1 for more details). UKBB, UK Biobank. Supplementary figure 2: Leave one out analysis. In order to test whether any individual SNPs were driving the association between JIA and (A) coronary artery disease, (B) strenuous sports, (C) paternal health and (D) number of non‐cancer illnesses, a leave on out analysis was conducted. Each row represents 2SMR analysis of JIA on the selected outcome using all SNPs in the JIA instrument, except the SNP listed on the y‐axis. The point represents the MR effect size (beta) and the bars represent 95% confidence intervals. The row in red represents the estimate when all SNPs are included in the analysis. [file ACR2-4-363-s003.pdf]

Supplementary Figures

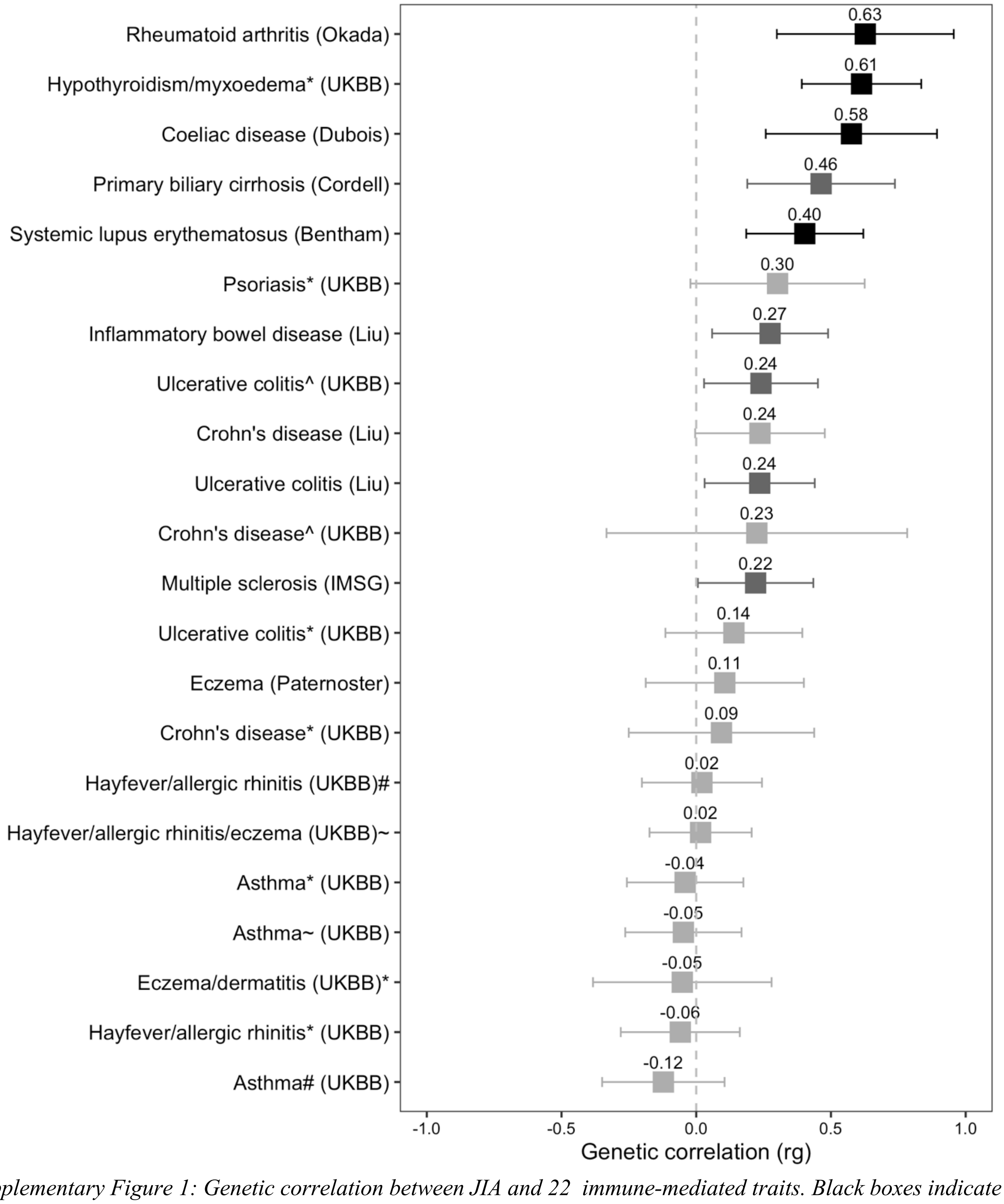

*Supplementary Figure 1: Genetic correlation between JIA and 22 immune-mediated traits. Black boxes indicate P_{adj} <0.05 after adjustment for multiple testing, dark grey boxes indicate unadjusted P <0.05 and light grey boxes indicate unadjusted P >0.05. Method of ascertainment of UK Biobank variables is indicated by superscript - *self-reported data, ^ICD-10 coded data, [#]doctor diagnosed data or ~diagnosed by doctor. Data source provided in parentheses for each trait (see Supplementary Table 1 for more details). UKBB, UK Biobank.*

Supplementary Figure 2

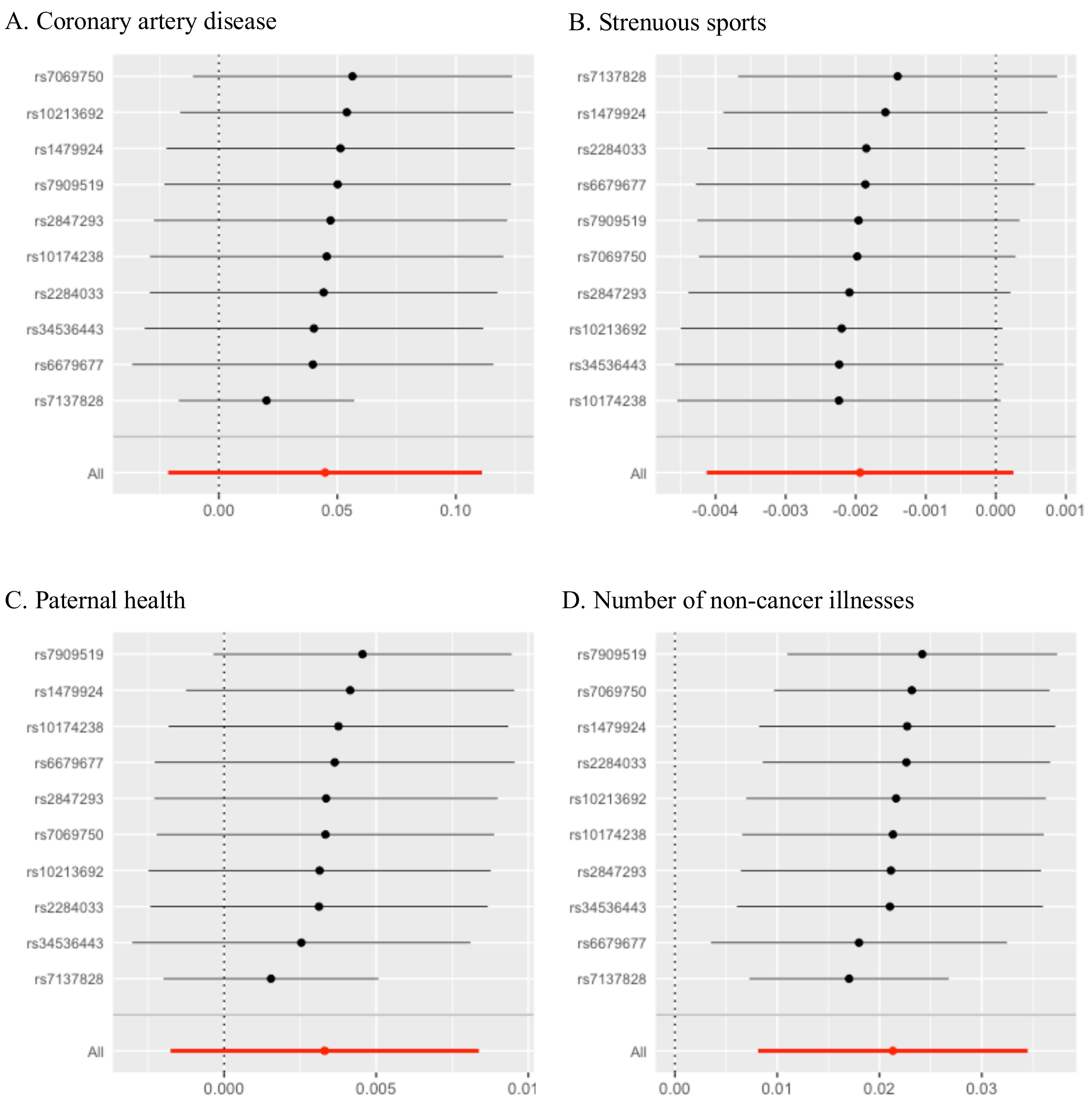

Supplementary figure 2: Leave one out analysis. In order to test whether any individual SNPs were driving the association between JIA and (A) coronary artery disease, (B) strenuous sports, (C) paternal health and (D) number of non-cancer illnesses, a leave on out analysis was conducted. Each row represents 2SMR analysis of JIA on the selected outcome using all SNPs in the JIA instrument, except the SNP listed on the y-axis. The point represents the MR effect size (beta) and the bars represent 95% confidence intervals. The row in red represents the estimate when all SNPs are included in the analysis.
