# Supplementary material for: Examining Health Outcomes in Juvenile Idiopathic Arthritis: A Genetic Epidemiology Study
Source: ACR Open Rheumatol. 2022 Jan 25;4(4):363–70. doi: 10.1002/acr2.11404 (PMC8992462; doi:10.1002/acr2.11404)
Supplement: Supplementary file 3 — Supplementary S1 Methods [file ACR2-4-363-s004.pdf]

Supplementary methods

Calculation of R^2 and F statistic

The strength of the genetic instrument is determined by the magnitude and precision of the single nucleotide polymorphism (SNP) -exposure associations. In a two sample Mendelian randomisation (MR) setting, weak instrument bias can attenuate the causal effect towards the null. The R^2 and F statistic for the SNP-exposure association provide an estimate of instrument strength; with higher values indicating a lower risk of weak instrument bias.[1] The F statistic was calculated for the MR instrument using $F = \left(\frac{N-K-1}{K}\right)\left(\frac{R^2}{1-R^2}\right)$, where N indicates sample size, K indicates number of SNPs in the instrument and R^2 indicates the proportion of the variance in the exposure explained by the instrument.

MR sensitivity analyses

The following methods were used to test and account for horizontal pleiotropy as they rely on one or more non-overlapping assumptions:

1. MR-Egger regression[2] is used to identify and account for unbalanced pleiotropy, and can provide an accurate estimate of causality even if all instrumental variables (IVs) are invalid. MR-Egger analysis uses the inverse variance of SNP-outcome associations as weights in a weighted linear regression of SNP-outcomes effects on SNP-exposure effects. The slope of this regression represents the casual effect estimate and, in the absence of pleiotropy, equals the IVW estimate. The intercept can be interpreted as the average pleiotropic effects of the IVs, with directional pleiotropy being indicated by an intercept other than zero. However the MR-Egger estimate assumes that there is no measurement error (NOME assumption) in the SNP-exposure association. The $I^2_{(GX)}$ statistic provides assessment of degree to which the NOME assumption is violated and thus relative bias in the MR-Egger estimate. Here $I^2_{(GX)}$ <90% is suggestive of substantial bias and addition adjustment to the MR-Egger estimate should be considered, such as SIMEX correction[3] (Supplementary Table 10).
2. The weighted median estimator[4] offers protection against invalid IVs/horizontal pleiotropy by providing a consistent estimate of causality when up to 50% of the weight comes from invalid IVs. It estimates the weighted median value of the Wald ratio.
3. MR-PRESSO[5] consists of three tests and is used to identify and correct for bias due to horizontal pleiotropy. The global test identifies whether horizontal pleiotropy is present. The outlier test returns an outlier-corrected causal estimate, accounting for horizontal pleiotropy. The distortion test estimates whether the outlier-corrected causal estimate is significantly different from the IVW estimate.
4. RadialMR[6] performs radial regression, allowing quantification of heterogeneity and identification of outliers using Cochran's Q-statistic. The reported estimate represents the IVW estimate obtained when the identified outliers are removed from the instrument. RadialMR was performed using an alpha level of 0·05 and modified second order weights.

Transformation of MR effect estimates

To aid the interpretability of the MR causal estimates (beta coefficients) a number of transformations have been applied:

1. For both binary outcomes derived from UK Biobank ("paternal health" and "strenuous sports") beta coefficient and standard error values were transformed from the risk difference scale to the log odds scale using $logOR = \frac{\beta}{(\mu(1-\mu))}$, where μ is the case fraction. Standard errors were similarly transformed using $se = \frac{se}{(\mu(1-\mu))}$.[7]
2. For the continuous outcome derived from UK Biobank ("number of non-cancer illnesses") the beta coefficient and standard error values were converted from per standard deviation increase to numeric increment by multiplying the beta coefficient and standard error values by the UK Biobank reported standard deviation unit (2·05694 for "number of non-cancer illnesses").
3. Following step 2, continuous outcomes ("number of non-cancer illnesses") are reported as beta coefficients and represent in increase in number of non-cancer illnesses per doubling odds of JIA.
4. For binary outcomes ("coronary artery disease", "paternal health" and "strenuous sports") the beta-coefficients represent the log odds ratio for each outcome per unit increase in the log OR of JIA risk. Since this is not intuitive, the betas were multiplied by 0·693 and then exponentiated to represent the odds ratio of each outcome per doubling in odds of JIA liability, as recommended for binary traits.[8]

Supplementary methods references

1. J Zheng, D Baird, MC Borges, J Bowden, G Hemani, P Haycock, et al. Recent Developments in Mendelian Randomization Studies. Curr Epidemiol Rep. 2017;4(4):330-45.
2. J Bowden, G Davey Smith and S Burgess. Mendelian randomization with invalid instruments: effect estimation and bias detection through Egger regression. International Journal of Epidemiology. 2015;44(2):512-25.
3. J Bowden, MF Del Greco, C Minelli, G Davey Smith, NA Sheehan and JR Thompson. Assessing the suitability of summary data for two-sample Mendelian randomization analyses using MR-Egger regression: the role of the I2 statistic. Int J Epidemiol. 2016;45(6):1961-74.
4. J Bowden, G Davey Smith, PC Haycock and S Burgess. Consistent Estimation in Mendelian Randomization with Some Invalid Instruments Using a Weighted Median Estimator. Genetic Epidemiology. 2016;40(4):304-14.
5. M Verbanck, CY Chen, B Neale and R Do. Detection of widespread horizontal pleiotropy in causal relationships inferred from Mendelian randomization between complex traits and diseases. Nat Genet. 2018;50(5):693-8.
6. J Bowden, W Spiller, MF Del Greco, N Sheehan, J Thompson, C Minelli, et al. Improving the visualization, interpretation and analysis of two-sample summary data Mendelian randomization via the Radial plot and Radial regression. Int J Epidemiol. 2018;47(4):1264-78.
7. PR Loh, G Tucker, BK Bulik-Sullivan, BJ Vilhjalmsson, HK Finucane, RM Salem, et al. Efficient Bayesian mixed-model analysis increases association power in large cohorts. Nat Genet. 2015;47(3):284-90.
8. S Burgess and JA Labrecque. Mendelian randomization with a binary exposure variable: interpretation and presentation of causal estimates. Eur J Epidemiol. 2018;33(10):947-52.
